# Chemical Composition and Biological Activities of Essential Oils of *Eremanthus erythropappus* (DC) McLeisch (Asteraceae)

**DOI:** 10.3390/molecules18089785

**Published:** 2013-08-16

**Authors:** Marcelo S. Silvério, Glauciemar Del-Vechio-Vieira, Míriam A. O. Pinto, Maria S. Alves, Orlando V. Sousa

**Affiliations:** Department of Pharmaceutical Sciences, Faculty of Pharmacy, Federal University of Juiz de Fora, São Pedro, Juiz de Fora, Minas Gerais 36036-330, Brazil; E-Mails: marcelosilverio@hotmail.com (M.S.S.); glauciemar@gmail.com (G.D.-V.-V.); miriamaop@yahoo.com.br (M.A.O.P.); alves_ms2005@yahoo.com.br (M.S.A.)

**Keywords:** *Eremanthus erythropappus*, essential oil, β-caryophyllene, germacrene-D, α-bisabolol, antimicrobial activity, antioxidant activity

## Abstract

The chemical composition of the essential oils obtained by hydrodistillation of different parts of *Eremanthus erythropappus*, including leaves, branches and inflorescences, was investigated by Gas Chromatography and Gas Chromatography/Mass Spectrometry. The antimicrobial activity of the oils was assessed by the disc diffusion and microdilution methods, while the antioxidant activity was evaluated by DPPH and reducing power tests. The main compounds found in the essential oils derived from the inflorescences and leaves were β-caryophyllene, germacrene-D, α-copaene and β-pinene. α-Bisabolol was the major component in the branches. The oils were active against *Staphylococcus aureus*, *Streptococcus pyogenes* and fungi, but not *Escherichia coli* and *Pseudomonas aeruginosa*. The MIC values ranged from 0.01 to 0.50 mg/mL. Using the DPPH test, the IC_50_ values ranged from 38.77 ± 0.76 to 102.24 ± 1.96 μg/mL, while the reducing power test produced IC_50_ values between 109.85 ± 1.68 and 169.53 ± 0.64 μg/mL. The results revealed that the *E. erythropappus* oils are new promising potential sources of antimicrobial and antioxidant compounds with good future practical applications for human health.

## 1. Introduction

Essential oils are lipophilic molecules responsible for flavours and fragrances and are obtained by steam distillation [1,2]. Most of them are mixtures of terpene and sesquiterpene hydrocarbons, phenylpropanoids and benzenoid oxygenated derivatives [2] that have been investigated for their antimicrobial and antioxidant properties [3,4,5,6]. These properties have been attributed to the chemical components, such as α-pinene, β-pinene, *p*-cymene and *γ*-terpinene [3,7], linalool [8], camphor, carvacrol, eugenol, geraniol, thymol, *trans*-cinnamaldehyde, *trans*-cinnamic acid and vanillic acid [9,10,11].

*Eremanthus erythropappus* (DC) McLeisch (Asteraceae) (*Vanillosmopsis erythropappa* Schultz-Bip), commonly known as candeia, is used in folk medicine as an antiphlogistic and antimicrobial agent [12]. The essential oil obtained from the wood of *E. erythropappus* contains α-bisabolol, costunolide and eremanthine [13,14,15], compounds with the cyclocostunolide and eremanthine skeleton [14,16], vanillosmin [17], 15-deoxygoyazenzolide [18] and lychnopholide [19]. The main compounds found in the leaves were β-pinene, β-caryophyllene, β-myrcene and germacrene-D [15,20,21]. α-Bisabolol, present in other members of the Asteraceae such as *Chamomilla recutita*, exhibits anti-inflammatory properties [22] and has been widely used in pharmaceutical and cosmetic products [12]. Previously, the essential oil obtained from the leaves of this species revealed antimicrobial [20], antinociceptive and anti-inflammatory activities [21]. The ethanol extract from *E. erythropappus* leaves also demonstrated these activities, as well as antiulcerogenic effects [23]. Moreover, the essential oil and β-bisabolene from this plant showed a potential to restore the effectiveness of ampicillin against resistant *Staphylococcus aureus* isolates [24].

Despite the exploration and trade of products containing essential oil from *E. erythropappus*, as well as chemical and biological studies, the antioxidant effect of the essential oils of this species has not been described. In this way, to complete and better understanding, essential oils from distinct parts as inflorescences, fresh leaves, dried leaves and branches of *E. erythropappus* were investigated for the chemical composition and antimicrobial and antioxidant activities.

## 2. Results and Discussion

The parts of *E. erythropappus* were found to contain 0.2%, 0.2%, 0.3% and 0.4% of essential oil for inflorescences, fresh leaves, dry leaves and branches, respectively. Twenty six compounds were identified in the essential oil from inflorescences and dry leaves, twenty five from fresh leaves, and three from branches, representing 99.8%, 99.6% and 100.0% of each total oil, respectively (Table 1). The compounds identified in the essential oils from *E. erythropappus* belong to mono and/or sesquiterpenes. The inflorescence oil was characterized by the abundance of β-caryophyllene (17.7%), germacrene-D (20.0%) and α-muurolol (10.0%), while β-caryophyllene (21.8% and 22.4%), germacrene-D (14.9% and 16.8%), α-copaene (10.2% and 8.7%) and β-pinene (8.6% and 9.1%) were the main constituents in the fresh and dry leaves, respectively. α-Bisabolol was the major component in the branch oil. It was observed that β-caryophyllene had a significant presence in the oils from inflorescences and fresh and dry leaves, thus being a true chemical marker of these essential oils from *E. erythropappus*. In addition, the chemical composition of essential oil from inflorescences has been described for the first time (Table 1).

**Table 1:** Main components of the essential oils from *Eremanthus erythropappus*.

Compound	Retention Index	Yield (%)				Method of identification
		Inflorescences	Fresh leaves	Dry leaves	Branches	
*Monoterpene hydrocarbons*		*6.1*	*14.6*	*17.3*		
α-pinene	936	0.9	2.0	3.0	−	RI, GC-MS, ST
β-pinene	975	4.0	8.6	9.1	−	RI, GC-MS, ST
myrcene	987	1.0	3.5	4.1	−	RI, GC-MS, ST
α-terpinene	1018	−	−	0.2	−	RI, GC-MS, ST
β-phellandrene	1031	−	0.5	0.6	−	RI, GC-MS, ST
limonene	1033	0.2	−	−	−	RI, GC-MS, ST
β-ocimene	1040	−	−	0.3	−	RI, GC-MS
*Oxygenated monoterpenes*		*0.8*	*0.9*	*1.3*		
linalool	1096	0.3	0.3	0.7	−	RI, GC-MS, ST
terpinen-4-ol	1174	0.1	0.3	0.2	−	RI, GC-MS, ST
α-terpineol	1188	0.4	0.3	0.4	−	RI, GC-MS, ST
*Sesquiterpene hydrocarbons*		*62.5*	*67.6*	*67.3*	*5.6*	
δ-elemene	1336	−	0.7	1.0	−	RI, GC-MS
α-cubebene	1348	−	0.5	0.2	−	RI, GC-MS
α-copaene	1377	−	10.2	8.7	−	RI, GC-MS
β-elemene	1390	7.4	4.1	2.5	5.6	RI, GC-MS
β-patchoulene	1380	0.4	−	−	−	RI, GC-MS
β-bourbonene	1384	−	1.1	1.1	−	RI, GC-MS
β-cubebene	1388	3.5	−	−	−	RI, GC-MS
cyperene	1401	5.7	−	−	−	RI, GC-MS
α-gurjunene	1409	0.6	0.8	0.7	−	RI, GC-MS
β-caryophyllene	1420	17.7	21.8	22.4	−	RI, GC-MS, ST
β-gurjunene	1434	1.2	−	−	−	RI, GC-MS
β-selinene	1489	3.1	5.0	5.5	−	RI, GC-MS
valencene	1496	4.1	0.5	0.5	−	RI, GC-MS
germacrene-D	1481	12.0	14.9	16.8	−	RI, GC-MS, ST
δ-cadinene	1522	6.8	8.0	7.9	−	RI, GC-MS
*Oxygenated sesquiterpenes*		*30.0*	*16.5*	*13.1*	*93.1*	
spathulenol	1575	1.2	4.3	2.1	−	RI, GC-MS
caryophyllene oxide	1578	6.0	4.0	4.5	−	RI, GC-MS, ST
humulene epoxide II	1605	5.1	−	−	−	RI, GC-MS
10- *epi*-γ-eudesmol	1609	1.3	−	−	−	RI, GC-MS
di- *epi*-cubenol	1616	−	1.1	3.1	−	RI, GC-MS
hinesol	1637	0.7	2.3	−	−	RI, GC-MS
cubenol	1642	0.5	−	−	−	RI, GC-MS
α-muurolol	1646	10.0	0.5	0.5	−	RI, GC-MS
α-cadinol	1653	5.2	2.2	1.5	−	RI, GC-MS
α-bisabolol	1685	−	2.1	1.4	93.1	RI, GC-MS, ST
*Total (%)*		99.4	99.6	99.0	98.7	
*Unidentified*		0.6	0.4	1.0	1.3	
*Yield (%)*		0.4	0.5	0.8	0.7	

Retention index on a HP-5 column with reference to *n*-alkanes [25]; MS, NIST and Wiley library spectra and the literature; RI: Retention index; ST: authentic standard compounds.

The essential oils were evaluated for antimicrobial activity against representatives of two Gram-positive and two Gram-negative bacterial strains. Using the agar diffusion method, the essential oils were active against *Staphylococcus aureus*, *Streptococcus pyogenes* and *Escherichia coli*, but not against *Pseudomonas aeruginosa*. The essential oils were also active against *Candida albicans* and *C. tropicalis* (Table 2), two important yeast strain representatives. The inhibition zones varied between 9 and 14 mm. The MIC values confirmed the activity against the tested microorganisms, as shown in Table 3. The MIC values of oils ranged from 0.01 to 0.31 mg/mL, while the MIC values for chloramphenicol varied from 1.25 to 5.00 mg/mL and for fluconazole was 0.015 mg/mL.

**Table 2:** Antimicrobial activity of the essential oils of *Eremanthus erythropappus*.

Microorganisms	Inhibition zone (mm)												
	Inflorescences (mg)			Fresh leaves (mg)			Dry leaves (mg)			Branches (mg)			Control
	5	10	20	5	10	20	5	10	20	5	10	20	
*S. aureus*	0	0	12	0	9	12	0	10	14	0	0	10	24
*S. pyogenes*	0	0	9	0	10	13	0	12	14	0	0	11	20
*E. coli*	0	0	0	0	0	0	0	0	0	0	0	0	26
*P. aeruginosa*	0	0	0	0	0	0	0	0	0	0	0	0	20
*C. albicans*	0	0	11	0	0	12	0	0	14	0	0	11	30
*C. tropicalis*	0	0	13	0	0	14	0	0	14	0	0	12	25

Experiments were done in triplicate and results were mean values. Control: chloramphenicol (1 μg/mL) for bacteria or fluconazole (20 μg/mL) for the yeast.

**Table 3:** Minimal inhibitory concentrations of the essential oils of *Eremanthus erythropappus*.

Microorganisms	MIC (μg/mL)				
	Inflorescences	Fresh leaves	Dry leaves	Branches	Control
*S. aureus*	125	310	310	500	2
*S. pyogenes*	40	20	10	250	4
*E. coli*	>1,000	>1,000	>1,000	>1,000	8
*P. aeruginosa*	>1,000	>1,000	>1,000	>1,000	>64
*C. albicans*	125	15	60	100	15
*C. tropicalis*	125	60	125	100	45

Experiments were done in triplicate and results were mean values.Control: Ampicillin (64–0.0625 μg/mL) for bacteria or fluconazole (64–0.0625 μg/mL) for the yeast.

Free radical scavenging activity results using DPPH presented as IC_50_ values were 38.77 ± 0.76, 61.61 ± 1.01, 49.06 ± 0.98 and 102.24 ± 1.96 μg/mL for inflorescences, fresh leaves, dried leaves and branches, respectively (Table 4). Reducing power of the oils is also shown in Table 4. As can be seen from the table, reducing power of the essential oils of the parts of *E. erythropappus* produced IC_50_ values that ranged from 109.85 ± 1.68 to 169.53 ± 0.64 μg/mL. According to the results, the most active sample was inflorescences, with an IC_50_ value of 109.85 ± 1.68 μg/mL.

It is important to emphasize that the antioxidant activity of the essential oils from *E. erythropappus* was described herein for the first time. Considering the composition, some variations were observed. For example, the β-pinene content in leaves was about two times lower than that previously reported [20,21]. Cyperene (5.7%), valencene (4.1%), caryophyllene oxide (6.0%), humulene epoxide II (4.1%) and others with smaller quantities found in inflorescences were not identified in a recently published study [15]. A possible explanation for this divergence is the collection period, as well as drying. These factors may have influenced the levels of oils and identified constituents [26]. Moreover, with the exception of hinesol (fresh leaves) and α-terpinene and β-ocimene (dried leaves), the chemical composition of these materials was similar (Table 1).

**Table 4:** Antioxidant activityof the essential oils of *E. erythropappus*.

Oils/Chemical	IC_50_ (μg/mL)	
	DPPH	Fe^+3^ Reducing Power
Inflorescences	38.77 ± 0.76	109.85 ± 1.68
Fresh leaves	61.61 ± 1.01	140.56 ± 0.51
Dry leaves	49.06 ± 0.98	135.23 ± 1.53
Branches	102.24 ± 1.96	169.53 ± 0.64
Rutin	5.07 ± 0.04	−
Ascorbic acid	−	63.41 ± 3.87

Each value in the table is represented as mean ± S.D. (n = 3). The values are significantly different (*p* < 0.05) −ANOVA and Tukey test.

The essential oil constituents could be considered as responsible for the antimicrobial and antioxidant activities [27,28]. Although they usually occur as complex mixtures, their action may generally be accounted for in terms of their major components. Antimicrobial and antioxidant activities verified in this study were also reported by essential oils of other plants belonging to the Asteraceae family [28,29,30,31]. Probably, similar components detected in our experiments could be responsible for these properties, such as for example germacrene-D, β-caryophyllene [31], linalool and valencene [32]. The antimicrobial activity of the oil could also be due to α-pinene and β-pinene [11]. Pinene-type monoterpene hydrocarbons (α-pinene and β-pinene) are chemicals well-known to have antimicrobial potential [33]. This potential also has been revealed for α-humulene, caryophyllene oxide, β-selinene and elemol, which were found in appreciable amounts in the investigated oils [20,21,22,24]. Furthermore, the antimicrobial activity of the essential oils from *E. erythropappus* is apparently related with terpenes type compounds such as myrcene and linalool (Table 1), since there is a relationship between the chemical structures of the most abundant oils and their antimicrobial activities. Being the most abundant component of the oil from the branches, α-bisabolol seems to have a significant contribution to the antimicrobial activity [24]. However, it should be considered that minor and major components, as well as possible interactions between the substances, could also contribute with the antimicrobial properties described in the present investigation. Essential oils containing terpenes are reported to possess antimicrobial activity [33], which is consistent with our present results.

Essential oils, as natural sources of antioxidants, have been evaluated for their activity as free radical scavengers. The DPPH is a stable free radical, which has been widely accepted as a tool for estimating free radical-scavenging activities of antioxidants [34]. The antioxidant activity is coupled to the redox properties, ability to scavenge a variety of reactive species such as superoxide, hydroxyl and peroxyl radicals and hypochlorous acid, singlet oxygen quenching and metal ion chelation [35]. In animal organs and tissue, oxidants can cause cellular damage through of the peroxidation of unsaturated fatty acids, denaturation of proteins and reaction with carbohydrates and nucleic acids [36,37]. These damages have been involved in the pathogenesis of several human disorders [36,37,38]. In this sense, natural products with properties against free-radicals could have great importance as therapeutic agents in different diseases, since are effectives as radical scavengers and inhibitors of lipid peroxidation [38]. The antioxidant activity found in our results might be attributed to the presence of high percentages of β-caryophyllene (17.7%, 21.8% and 22.4%), germacrene-D (14.9%, 16.8% and 20.0%), α-copaene (10.2% and 8.7%), β-pinene (8.6% and 9.1%), α-muurolol (10.0%) and α-bisabolol (93.1%) (Table 1). α-Terpineol and α-pinene previously found in *E. erythropappus* oil [20,21] have inherent antioxidant activity attributed mainly to terpinene and terpinolene contents. In general, Asteraceae species are well documented as natural antioxidants [39] but to our knowledge, no studies were conducted on essential oils of *E. erythropappus*.

Although the mechanism of action of terpenes is not fully understood, it is thought to involve membrane disruption by the lipophilic compounds [40]. The results may justify the use of *E. erythropappus* in traditional medicine. Therefore, the essential oils from this species are potential candidates to be used as antimicrobial and antioxidant agents in new drugs for therapy of infectious diseases and human disorders. The synergistic effects of the chemical compounds of the essential oils should be taken into consideration for the antimicrobial and antioxidant activities. Further toxicological and clinical studies are required to prove the safety of the oil as a medicine.

## 3. Experimental Section

### 3.1. Plant Material

Plant material from different parts of *E. erythropappus* (DC) McLeisch (Asteraceae) was collected in March 2008, at the city of Juiz de Fora, Minas Gerais State, southeast region of Brazil at coordinates 43° 21' 01'' W (longitude) and 21° 45' 51'' S (latitude). The species was identified by Dr. Fátima Regina Gonçalves Salimena, and a voucher specimen (CESJ n° 25363) was deposited in the Herbarium of the Federal University of Juiz de Fora (UFJF), Juiz de Fora, MG, Brazil.

### 3.2. Isolation of the Essential Oils

Inflorescences, dry and fresh leaves, and branches of *E. erythropappus* were separated and triturated. After this procedure, 200 g of each sample were subjected to hydrodistillation for 2 h in a Clevenger-type apparatus [41]. The essential oils were removed from the surface of the water. The oils were dried over anhydrous sodium sulphate. The samples were sealed and kept in dark glass vials under refrigeration for further analysis.

### 3.3. Gas Chromatographic Analysis

Capillary gas chromatography was performed using a Hewlett-Packard 6890 gas chromatograph under the following conditions: fused silica capillary column HP-5 (5% diphenyl and 95% dimethylpolysyloxane, 60 m × 0.25 mm, 0.25 μm film thickness); helium as carrier gas (1 mL/min); and temperature programming from 70 to 290 °C (2 °C/min); injector temperature 270 °C and detector temperature 300 °C; injected volume 0.6 μL diluted in hexane (1:10).

### 3.4. Gas Chromatography/Mass Spectrometry Analysis (GC/MS)

The GC/MS analysis of the oils were performed on a Hewlett-Packard HP 6890 gas chromatograph equipped with a ZB-5MS column (30 m × 0.25 mm × 0.25 μm film thickness) and a FID detector was used for quantitatively determining oil components. Oven temperature was programmed as follows: 50 °C for 2 min, rising to 250 °C at 5 °C min^−1^. Injector temperature: 270 °C. Carrier gas: He with a flow rate of 1 mL/min^−1^. Detector temperature: 250 °C, split ratio: 1:10. Identification of the oil components was based on their retention indices and mass spectra, obtained from GC-MS analysis on a Hewlett-Packard HP 6890/HP5973 equipped with a DB-5 fused silica capillary column (30 m × 0.25 mm × 0.25 μm film thickness). The GC analysis parameters listed above and the MS were obtained (full scan mode: scan time: 0.3 s, mass range was *m/z* 30–500) in the EI mode at 70 eV; injected volume 0.6 μL diluted in hexane (1:10). All data were the average of triplicate analyses.

### 3.5. Component Identification

Identification of the essential oil constituents was based on comparisons of retention index (RI) (determined relatively to the retention times of a *n*-alkanes series), retention times (RT), and mass spectra with those obtained from authentic standards and/or the NIST and Wiley libraries spectra, and relevant literature [25].

### 3.6. Microbial Strains

Strains of bacteria and fungi were obtained from ATCC (American Type Culture Collection, Rockville, MD, USA). The studied microbial strains include *Staphylococcus aureus* (ATCC 6538), *Streptococcus pyogenes* (ATCC 19615), *Escherichia coli* (ATCC 8739), *Pseudomonas aeruginosa* (ATCC 15442), *Candida albicans* (ATCC 10231) and *Candida tropicalis* (ATCC 13803).

### 3.7. Screening for Antimicrobial Activity

The antimicrobial activity was tested through the agar diffusion method [42]. Mueller Hinton Agar was used as the standard test medium for bacteria, with the exception of *Streptococcus pyogenes* (in this case, the Mueller Hinton Agar was supplemented with Sheep Blood at 8%), and Sabouraud Agar for yeast. Overnight broth cultures were prepared, adjusted in peptone-physiological salt solution (1 g peptone and 8.5 g/L NaCl) to yield approximately 10^6^ bacteria/mL and 10^5^ conidia/mL. The agar plates were prepared in 90 mm Petri dishes with 22 mL of agar medium, giving a final depth of 4 mm. Sterile cellulose discs, diameter 6 mm, were placed on the inoculated agar surfaces with 100 μL of diluted oil in hexane. Each 100 μL had 5, 10 or 20 mg of essential oil. Hexane (100 μL) was used as negative control. All plates were aerobically incubated at 35 °C for 18–24 h (bacteria) and at 22 °C for 48 h (yeast). The antimicrobial activity was estimated by measuring the radius of the inhibition zone (mm). Each test was performed in triplicate and the results were shown as means. 20 μL of chloramphenicol (1 mg/mL) for bacteria and 20 μL of fluconazole (20 μg/mL) for the yeast were used as positive controls.

### 3.8. Determination of the Minimum Inhibitory Concentration

The broth microdilution method recommended by the Clinical and Laboratory Standards Institute was used to determine the minimum inhibitory concentration (MIC) values [43,44]. Antibacterial activity tests were performed in Mueller-Hinton broth and for antifungal tests, RPMI-1640 medium with L-glutamine, buffered with MOPS buffer was used. The inoculum densities were approximately 5 × 10^5^ CFU/mL and 0.5–2.5 × 10^3^ CFU/mL for bacteria and fungi, respectively. Each essential oil was dissolved in Tween 80 and sterile distilled water. Final two fold concentrations were prepared in the wells of the microtiter plates, between 1,000–10 μg/mL. Ampicillin and fluconazole were used as reference antibiotics for bacteria and fungi, respectively (64–0.0625 μg/mL). Microtiter plates were incubated at 35 °C for 18–24 h for bacteria and at 22 °C for 48 h for yeast. After the incubation period, MIC values were defined as the lowest concentration of the oils that inhibits the visible growth of microorganisms.

### 3.9. Free Radical Scavenging Activity Determination

Sample stock solutions (1.0 mg/mL) were diluted to final concentrations of 250, 125, 50, 25, 10 and 5 mg/mL, in ethanol. One mL of a 0.3 mM DPPH ethanol solution was added to 2.5 mL of sample solutions of different concentrations, and allowed to react at room temperature [45]. After 30 min, the absorbance values were measured at 518 nm and converted into the percentage antioxidant activity (AA) using the following formula:

AA% = 100 − {[(Abs_sample_ − Abs_blank_) × 100]/Abs_control_}

Ethanol (1.0 mL) plus essential oils solution (2.5 mL) was used as a blank. DPPH solution (1.0 mL; 0.3 mM) plus ethanol (2.5 mL) was used as a negative control. The positive controls were those using the standard solutions. The IC_50_ values were calculated by linear regression of plots where the abscissa represented the concentration of tested plant extracts and the ordinate the average percent of antioxidant activity from three separate tests.

### 3.10. Reducing Power Assay

The reducing power of the essentials was determined according to the procedure described by Sutana *et al.* [46]. Concentrated extract (2.5–10.0 mg) was mixed with sodium phosphate buffer (5.0 mL, 0.2 M, pH 6.6) and potassium ferricyanide (5.0 mL, 1.0%); the mixture was incubated at 50 °C for 20 min. Then 10% trichloroacetic acid (5 mL) was added and the mixture centrifuged at 980 g for 10 min at 5 °C in a refrigerated centrifuge (SPLabor, Presidente Prudente, Brazil). The upper layer of the solution (5.0 mL) was decanted and diluted with 5.0 mL of distilled water and ferric chloride (1.0 mL, 0.1%), and absorbance read at 700 nm using a spectrophotometer (SHIMADZU^®^, UV-1800, Tokyo, Japan). Ascorbic acid was used as standard. All samples were analyzed thrice and the results averaged. The IC_50_ values were calculated by linear regression of plots where the abscissa represented the concentration of tested oil essential oils and the ordinate the average percent of antioxidant activity from three separate tests.

### 3.11. Statistical Analysis

Data were expressed as mean ± S.D. Statistical significance was analysed by the one-way analysis of variance followed by the Tukey test. *p* values below 0.05 were considered significant.

## 4. Conclusions

To the best of our knowledge, this is the first study to provide data on the antimicrobial and antioxidant activities of the essential oils from *E. erythropappus* that confirm its medicinal properties. For the first time, we demonstrate that the essential oils of this species exhibit antioxidant activity. The results obtained in this study show that the essential oils of *E. erythropappus* may be a potential source of natural antioxidants and antimicrobial agents. However, further studies need to be conducted to understand the mechanism of the activity and obtain more information on the safety and toxicity of the oils. 
